# Proceedings of the third CCP-EM Spring Symposium

**DOI:** 10.1107/S2059798318008239

**Published:** 2018-06-01

**Authors:** Tom Burnley, Paula da Fonseca

**Affiliations:** a Scientific Computing Department, Science and Technology Facilities Council, Research Complex at Harwell, Didcot OX11 0FA, UK; b MRC Laboratory of Molecular Biology, Francis Crick Avenue, Cambridge Biomedical Campus, Cambridge CB2 0QH, UK

**Keywords:** CCP-EM Spring Symposium, cryo-EM

## Abstract

An introduction to the proceedings of the third CCP-EM Spring Symposium.

This special issue covers the proceedings of the third CCP-EM Spring Symposium. The 2017 Symposium highlighted cutting-edge developments in macromolecular cryo-electron microscopy with a particular focus on methods with exemplar applications. This is a tremendously exciting time for life-sciences cryo-EM, crowned by the award of the Nobel Prize for Chemistry in 2017 to Jacques Dubochet, Joachim Frank and Richard Henderson for ‘developing cryo-electron microscopy for the high-resolution structure determination of biomolecules in solution’.

In 2017 the Spring Symposium was twinned with the opening of the electron Bio-Imaging Centre (eBIC) located at the Diamond Light Source (DLS) in Oxfordshire, UK. This is the national centre for biological cryo-EM in the UK and has the same open-access philosophy as its host synchrotron. This model has successfully provided access to world-class X-ray facilities and has been instrumental in the growth and success of the macromolecular crystallography community. Providing similar facilities for cryo-EM is an important development in the democratization of the technique: providing access to both state-of-the-art equipment and experienced operating staff. Since the ‘resolution revolution’ cryo-EM has witnessed a huge upsurge in usage, attracting structural biologists from varied domains, both from academia and from industry. Providing for these new researchers will help fuel further advances in the field.

From the accolades, the technological advances and the increase in userbase, cryo-EM is now witnessing progress towards mainstream usage and automation. For this there are still many challenges to address, from sample preparation through to high-resolution model building and validation. Several articles in this issue tackle some of these fundamental topics, whilst others highlight successful applications.

The issue has an ensemble of articles describing new methods for high-resolution model building and refinement. These take inspiration from techniques long established for building atomic models using X-ray diffraction data. However, it should be noted that cryo-EM and X-ray crystallography data have significant differences and these new methods have been optimized for the idiosyncrasies of cryo-EM. One such difference is the practice of sharpening (or blurring) of cryo-EM maps to maximize interpretability. Owing to the combination of errors during the reconstruction process it is not currently possible to determine the optimal sharpening parameters analytically during reconstruction, and these are therefore estimated empirically post-processing. In this issue Tom Terwilliger *et al*. present methods to automate map sharpening, based on defining and quantifying ideal map qualities (detail and connectivity) and maximizing these desired properties *via* sharpening. Pavel Afonine and co-authors, and Rob Nicholls and co-authors both present new tools for automated refinement of EM-derived atomic models, but interestingly take divergent approaches: the former primarily uses real-space methods whilst the latter uses reciprocal space. Tristan Croll presents a third method, interactive modelling, in his article on *ISOLDE*. This approach combines, with user guidance, molecular dynamics and molecular graphics to refine/rebuild models.

The possibility of high-resolution atomic model building owes much to the advances in detector technology that have greatly enhanced the resolution of three-dimensional maps produced from image reconstruction, and these improvements in signal to noise also benefit electron diffraction. A particularly useful application of this method is for recording electron-diffraction data from nanosized crystals of biological specimens, too small for measurement by traditional X-ray diffraction. Max Clabbers and co-workers describe the adaptation of the *DIALS* software: from processing X-ray diffraction images to electron-diffraction images. In doing so they compare and contrast the methods and highlight key differences. Of course one key benefit of traditional single-particle cryo-EM is that it is not dependant on the crystallization of the target molecule. However, sample preparation for cryo-EM is non-trivial and can provide its own pitfalls and bottlenecks. This is addressed in the final methods article which comes from Ieva Drulyte and colleagues. This practical paper details the strategies they have evolved at their facility for optimal sample preparation.

In addition to the articles on recent developments on cryo-EM methods described above, two articles focus on their application to study particular biological systems. In their article, Moores and colleagues describe electron tomography studies of microtubules polymerized *in vitro* and compare those with *in vivo* assemblies, and how the structural information obtained contributes to the unveiling of the microtubule dynamic nature. Rossmann and Beeby review how electron tomography and subtomogram averaging is contributing to the understanding of bacterial flagellar motors, and how these studies provide insights into their evolution.

Together with our fellow Editor Randy Read we would like to thank all contributors to this second edition and hope for many more such proceedings in the future. Furthermore, since its outset CCP-EM has been primarily funded by the MRC and hosted by the STFC. We would like to thank both research councils for their continued support.

